# User Experience of a Canadian Web Application for On-Demand Preventive Health Care: Cross-Sectional Online Survey Study

**DOI:** 10.2196/90726

**Published:** 2026-10-27

**Authors:** Sophia Siedlikowski, Cayden Peixoto, Lise M Bjerre, Cleo Mavriplis

**Affiliations:** 1 Faculty of Medicine University of Ottawa Ottawa, ON Canada; 2 School of Epidemiology and Public Health Faculty of Medicine University of Ottawa Ottawa, ON Canada; 3 Department of Family Medicine University of Ottawa Ottawa, ON Canada; 4 Institut du Savoir Montfort Ottawa, ON Canada; 5 Department of Family Medicine McGill University Montreal, QC Canada

**Keywords:** medical informatics, preventive health services, digital health interventions, web applications, primary health care

## Abstract

**Background:**

Amid the current primary care crisis, Canadians are increasingly turning to online resources, where misinformation is common. Launched in 2023, the web application Icanbewell provides free, reliable, evidence-based information on preventive health care.

**Objective:**

This study aimed to evaluate the user experience of the Icanbewell web application.

**Methods:**

We conducted a cross-sectional online survey in Canada. Participants were members of the public aged at least 18 years and health care providers residing in Canada who had internet access. A 9-item online survey was made available within the Icanbewell web application from July 8, 2024, to August 7, 2025. It invited users to rate their satisfaction with the app, suggest improvements, and describe what they learned from it. The single Likert-scale question was analyzed using summary descriptive statistics, and the short-answer questions were analyzed using qualitative content analysis.

**Results:**

Of the 69 users who responded to the Icanbewell survey, 80% (n=55) were satisfied with the app, 9% (n=6) were dissatisfied, and 10% (n=7) were neither satisfied nor dissatisfied. Satisfaction ranged from 78.9% to 90.9% across participant gender, status as a health care provider or member of the public, language, and whether the respondent was attached to a primary care provider. A total of 46 (67%) respondents provided suggestions to improve the Icanbewell web application by optimizing the interface and adding medical content beyond prevention. Finally, 42% (29/69) of respondents indicated that they learned new information from using the Icanbewell app, including details about Canadian prevention guidelines, how to access various health resources, and general medical information on health and symptoms.

**Conclusions:**

This study demonstrated user satisfaction and engagement with the app. Subsequent work should prioritize representative sampling to gather feedback from individuals not attached to a primary care provider, younger people, health care providers, and people in geographically diverse locations. Our results highlight an ongoing need for accessible, trustworthy, evidence-based prevention resources, along with support for clinicians in disseminating them to the public. Future involvement of patient partners in the design and evaluation of the web application will be important to ensure its inclusivity and relevance.

## Introduction

It is estimated that 50% to 80% of all chronic diseases could be prevented [1,2]. In the 2025 OurCare survey on the public’s perception of the Canadian health care system, participants identified prevention as a top priority within the health care system. Specifically, they “underscored the importance of a prevention-focused, wellness-promoting primary care system that aims to address health problems before they occur” [3]. Prevention requires access to knowledge and resources, and primary care providers play an important role in the dissemination of this knowledge to the public. However, 6.5 million Canadians do not currently have a primary care provider [4]. Consequently, Canadians are turning more frequently to online sources, including mobile apps, for health information [5]. Most online health apps available to the public, however, are not evidence-based, do not have a medical expert involved, and provide no assurances about the secure collection and use of personal information [6-8]. Furthermore, in 2025, the Canadian Medical Association published an alarming report, revealing that 43% of Canadians are susceptible to believing health misinformation [5]. An evidence-based Canadian online resource for preventive care developed by health care providers could be a promising solution to this problem.

Since the pandemic, digital health interventions (DHIs), such as apps, sensors, websites, and virtual care models, have increasingly been developed to address the need for rapid access to health information among the public [9,10]. The evidence on the effectiveness and safety of these DHIs is complex and still emerging [11]. A 2024 overview of systematic and scoping reviews focusing on existing DHIs in primary care, including preventive care tools, showed mixed results regarding the impact of these interventions on health outcomes [12]. Other systematic reviews have, however, demonstrated that certain DHIs helped reduce adverse cardiovascular outcomes, had positive impacts on cardiovascular risk factors, and showed favorable effects on costs and health outcomes in general [13-15].

After publishing a review article on Canadian preventive guidelines in 2016, Shimizu et al [16] (CM was a coauthor) received feedback from physicians requesting an electronic resource that could be easily updated with changing evidence, as well as a tool to educate patients. To help fill this gap in rapid access to reliable information, CM and her team created the free, bilingual Canadian web application Icanbewell (Choixsante in French). The web application, endorsed by the College of Family Physicians of Canada, offers 2 interfaces: 1 for members of the public and 1 for health care providers, in both French and English. The web application provides free, reliable, personalized preventive health information when age and gender are entered and includes information for gender diverse people [17]. For example, the web application provides age-based information on screening for chronic diseases, tips for skin cancer prevention, and recommendations for healthy nutrition. This objective aligns with the Quintuple Aim framework for improvement of population health and advancement of health equity [18].

Prior to the present study, a small feasibility and acceptability study found the web application to be generally easy to use and identified areas for improvement in its clarity, personalization, and Canadian relevance [19]. These findings informed refinements to the web application’s content and functionality prior to its public launch. Since the public launch of the web application in 2023, however, the Icanbewell web application user experience has not been formally evaluated. This study therefore aimed to evaluate the user experience and satisfaction of health care providers and members of the public.

A better understanding of who is using Icanbewell and how users think it can be improved will allow the team to make appropriate modifications, leading to stronger user satisfaction and health literacy. Systematically collecting user feedback through the integration of a permanent feedback link in the user interface can facilitate such an evaluation [20]. To our knowledge, this is the first study to evaluate a Canadian, bilingual, evidence-based web application focused on disease prevention.

## Methods

### Study Design and Context

We used a cross-sectional survey design to capture user perspectives on the Icanbewell web application from both health care providers and the general population. The study was conducted online in Canada from July 8, 2024, to August 7, 2025. We followed the CHERRIES (Checklist for Reporting Results of Internet E-Surveys) guidelines [21] for reporting online surveys (Multimedia Appendix 1).

### Ethical Considerations

The Institut du Savoir Montfort Research Ethics Board approved this study (24-25-06-012). Participants provided informed consent before completing the survey, and responses were anonymous. Participants were not compensated for participating.

### Data Collection

Convenience sampling was used to recruit participants for this study. The research team distributed posters with QR codes about the free web application at various primary care clinics and community centers in Ottawa. The team also shared information through online newsletters and social media channels. Users additionally discovered the web application by word of mouth. The authors attempted to share news of the study informally with colleagues in other provinces.

Upon accessing the Icanbewell website and after clicking on a topic, all application users were invited to participate in a 2-minute survey (Multimedia Appendix 2) offered in either French or English through a pop-up message. Consent was obtained by reading the invitation to the survey, which specified the purpose of the survey and stated that responses would be completely anonymous. The web application was designed so that the analytics did not collect IP addresses from users. There was no possibility of linking survey responses back to users. There were no incentives provided for participation in the study.

The 9-item online survey on user satisfaction and demographics (Multimedia Appendix 3) was made available from July 8, 2024, to August 7, 2025, to collect responses from adult users aged ≥18 years. Age was not verified, as the web application relied on users self-identifying as adults aged ≥18 years.

To measure satisfaction, users were prompted to answer 1 Likert-scale question and were then given 1 open-text question in which they could elaborate on suggestions for improvement (Multimedia Appendix 3). Users were also asked a second open-text question about what they learned from using the app. We used these 2 brief open-text responses to allow efficient data collection across our sample.

Study data were collected and managed using REDCap (Vanderbilt University) electronic data capture tools hosted at the Institut du Savoir Montfort [22,23]. Survey respondents included members of the public and health care providers.

### Data Analysis

#### Statistical Analysis

Responses from the 1 Likert-scale question on satisfaction were analyzed using summary statistics. We combined satisfaction categories to obtain broader yet still valuable insights into user perspectives. We therefore merged the “somewhat satisfied” and “very satisfied” data into the category “satisfied,” and also merged the “neither dissatisfied nor satisfied,” “somewhat dissatisfied,” and “very dissatisfied” data into the category “dissatisfied.” The inclusion of the neutral response option in the “dissatisfied” category was done to prevent bias toward user satisfaction.

We then analyzed user satisfaction across demographic subgroups. Proportions are reported with 95% CIs calculated by the Wilson score method. If a user opened the survey and did not respond to the question on user satisfaction, we subsequently removed the response from the analysis of user satisfaction across subgroups.

In an attempt to increase statistical power given an anticipated smaller sample, even if a participant did not complete the 9-item survey in its entirety (ie, missed at least 1 sociodemographic question), we retained their responses for the remaining completed items. We indicated any missing response by “not specified” in our results.

#### Qualitative Analysis

For the qualitative data, we ensured that our analysis met qualitative research standards as described by Lincoln and Guba [24], aiming for dependability, credibility, transferability, and confirmability. We used an inductive, conventional content analysis approach to analyze the responses to the following two short-answer questions asked in the survey (Multimedia Appendix 3):

1. Please give any suggestions to improve this app.
2. What did you learn from this app?

Responses in French were translated into English by a bilingual author (SS) before analysis. Translation accuracy was reviewed by all team members and verified with DeepL (DeepL SE) [25]. In our final reporting of results, we included the English translations of any French quotations.

To analyze the qualitative data, 2 team members (SS and CP) independently reviewed all free-text responses and generated preliminary subthemes for each question. There was no formal codebook given the limited number of questions and the depth of the answers. The 2 coders then met on 2 occasions to compare subthemes and reach consensus. Subthemes were named and refined iteratively through discussion with a senior team member (CM). Any disagreements were resolved through further discussion. All authors subsequently reviewed and agreed on the full set of finalized subthemes.

## Results

### Overview

A total of 69 users responded to the Icanbewell survey between July 8, 2024, and August 7, 2025. Unforeseen technical errors occurred during the study period, causing inconsistencies in web application analytics tracking. Consequently, data on precise numbers of users who were exposed to the web application and survey throughout the study period were unavailable. Therefore, we cannot report a response rate. Multimedia Appendix 4 shows the web application analytics we were able to retrieve. Of the 69 respondents to the study survey, only 4 participants did not complete the survey in its entirety (ie, missed at least 1 sociodemographic question). All responses were retained for completed survey items.

Descriptive statistics of the study population are provided in Table 1. Most survey respondents were English-speaking (57/69, 83%), women (49/69, 71%), members of the public (55/69, 80%), aged >50 years (51/69, 74%), living in Ontario (41/69, 59%), and attached to a primary care provider (59/69, 86%).

A total of 55 (80%) respondents were satisfied with the app, 6 (9%) were dissatisfied, and 7 (10%) were neither satisfied nor dissatisfied (Table 2). One user did not specify their level of satisfaction and was therefore removed from the analysis of satisfaction among groups.

Satisfaction was high and similar across subgroups (Table 3), ranging from 79.6% (43/54; 95% CI 67.1-88.2) among members of the public to 90.9% (10/11; 95% CI 62.3-98.4) among health care professionals, 78.9% among men (15/19; 95% CI 56.7-91.5) and 81% (39/48; 95% CI 68.1-89.8) among women, with near-identical estimates by language (English: 45/56, 80.4%, 95% CI 68.2-88.7; French: 8/10, 80%, 95% CI 49.0-94.3) and by attachment to a primary care provider (attached: 47/58, 81%, 95% CI 69.1-89.1; not attached: 8/10, 80%, 95% CI 49.0-94.3). The qualitative results revealed a diversity of responses to the 2 questions. The 2 main themes corresponded to the question prompts: suggestions for improvement and what users learned.

**Table 1:** Demographic characteristics of users (N=69).

Characteristics			Users, n (%)
**Age (years)**			
	18-29	4 (6)	
	30-49	13 (19)	
	50-64	31 (45)	
	≥65	20 (29)	
	Not specified	1 (1)	
**Gender**			
	Woman	49 (71)	
	Man	19 (28)	
	Nonbinary	1 (1)	
**Participant type**			
	Member of the public	55 (80)	
	Health care provider	11 (16)	
	Not specified	3 (4)	
**Attachment to doctor of medicine or nurse practitioner**			
	Yes	59 (86)	
	No	10 (14)	
**Geographic location**			
	Ontario	41 (59)	
	Quebec	20 (29)	
	Nova Scotia	4 (6)	
	British Columbia	1 (1)	
	Manitoba	1 (1)	
	Nunavut	1 (1)	
	Not specified	1 (1)	
**Preferred language**			
	English	57 (83)	
	French	10 (14)	
	German	1 (1)	
	Not specified	1 (1)	

**Table 2:** Overall satisfaction with Icanbewell among survey respondents (N=69).

Levels of satisfaction	Respondents, n (%)
Satisfied	55 (80)
Neither dissatisfied nor satisfied	7 (10)
Dissatisfied	6 (9)
Not specified	1 (1)

**Table 3:** Proportion of respondents reporting satisfaction with Icanbewell, by subgroup, with 95% CIs.

Variables^a^ and categories		Total respondents, N	Respondents satisfied^b^, % (n/N)	Satisfied 95% CI^c^ (%)
**Status (n=65)**				
	Health care provider	11	90.9 (10/11)	62.3-98.4
	Member of the public	54	79.6 (43/54)	67.1-88.2
**Language (n=66)**				
	English	56	80.4 (45/56)	68.2-88.7
	French	10	80 (8/10)	49.0-94.3
**Attachment to a primary care provider (n=68)**				
	Attached	58	81 (47/58)	69.1-89.1
	Not attached	10	80 (8/10)	49.0-94.3
**Gender (n=67)**				
	Woman	48	81.3 (39/48)	68.1-89.9
	Man	19	78.9 (15/19)	56.7-91.5

^a^Denominators vary (65-68) because of missing data for individual characteristics.

^b^Respondents satisfied are the proportion of users who were satisfied with the web app within one particular subgroup. The denominator for this ratio is provided in the second column, titled “Total respondents,” which is the N for each subgroup.

^c^CIs were calculated using the Wilson score method.

### Suggestions for Improvement

A total of 46 (67%) respondents provided suggestions to improve Icanbewell (Table 4). Three subthemes under this theme of improvement emerged from the analysis: (1) increasing access and optimizing the interface, (2) requests to add or modify the app’s medical content beyond prevention (ie, symptoms and treatment), and (3) general positive feedback on their user experience. Overall, users seemed to appreciate the “one-stop shop” concept of the web application as a way of keeping up to date with health information from multiple sources that are consolidated into 1 platform.

**Table 4:** Qualitative data analysis theme 1: suggestions for improvement.

Subthemes	Examples	Sample quote^a^
Access and user interface	Convert to a downloadable app Use a more inclusive body image: a more vague anatomical drawing would appeal to a broader audience Provide more guidance to users on how the app is organized and how to use it to decrease the sense of overwhelm Add a chatbot component and search box	“Ability to save an article then retrieve a list of saved or favourites. Also a quick feature to share a resource with someone”
Request to modify or add medical content	Add information on more medical conditions, suggestions for treatment at home, and when to seek care for spinal health, perimenopause, gender dysphoria and gender-affirming care, and mental health Ensure that the information reflects all provinces and territories	“Many older people including me have back problems - it would be helpful to have a section on spinal health & resources.”
General positive feedback	Useful and comprehensive resource for people who are motivated to stay healthy Satisfactory definitions and explanations of gender identities Lots of useful information consolidated in 1 place Appreciation for evidence-based medical information	“It’s really quite interesting because this site empowers patients to modify their lifestyle habits, whether in relation to breast cancer, osteoporosis, etc.”^b^

^a^Quotes are reported verbatim as written by respondents. Spelling errors were corrected for ease of comprehension.

^b^This quote is the authors’ English translation of a French quote, verified using DeepL [25].

### What Users Learned

A total of 29 (42%) respondents indicated that they learned new information from using the Icanbewell web application (Table 5). Three subthemes emerged from this main theme. Users learned about: (1) Canadian prevention guideline specifics, (2) how to access various health resources, and (3) general medical information on health and symptoms.

**Table 5:** Qualitative data analysis theme 2: what users learned.

Subthemes	Examples	Sample quote^a^
Canadian prevention guidelines	When and how frequently to test for colorectal cancer, lung cancer, prostate cancer, and osteoporosis Recommended calcium intake Risk of developing breast cancer or cardiovascular disease	“I learned I am too old for a colorectal cancer test.”
How to access health resources	How to get care without a primary care provider New health information websites and other resources	“...that there exists a certain number of specialized governmental and medical society guidelines.”^b^
General medical information	How to take blood pressure Dementia symptoms Dietary recommendations	“I began to learn about the differences between dementia and normal forgetfulness associated with aging.”

^a^Quotes are reported verbatim as written by respondents. Spelling errors were corrected for ease of comprehension.

^b^This quote is the authors’ English translation of a French quote, verified using DeepL [25].

## Discussion

### Principal Findings

Our study evaluated the user experience of the Icanbewell web application, a Canadian preventive DHI, using a cross-sectional survey design. This study comes at a pivotal time, as most Canadians want to prevent health problems before they occur [3]. Overall, the results of this initial evaluation of the Icanbewell application user experience demonstrate that respondents valued the application as a digital health information tool, with many engaging with the application’s preventive health content. Participants in the present study often reported learning when screening tests are appropriate according to Canadian guidelines. Importantly, our results suggest that users are interested in disease prevention information.

Most survey respondents (55/69, 80%) were satisfied with their experience using the Icanbewell app, with many highlighting the importance of having access through the application to a central repository of Canadian health guidelines created by Canadian health care providers and scientists. Moreover, 67% (46/69) of respondents provided suggestions to improve the application. One of the most frequent suggestions from respondents was to include additional medical content in the web application, beyond prevention. This desire for more information aligns with the outcomes of a recent Canadian Medical Association survey, which found that Canadians are increasingly searching online for medical information when they have difficulty accessing care in person [5]. Other important suggestions by survey respondents included improvements to the user interface of the web application, particularly in user navigation and content presentation.

User suggestions to add supplementary medical content highlight the challenges of presenting users with complete but not overwhelming health information, which can also be personalized for individual needs. Incorporating AI in future versions of the application may help improve the user experience in this regard. For example, AI could provide tailored answers to user questions and direct them to pertinent information within the app. This would lessen the “overwhelmed” feeling, which some participants reported. However, any use of AI in the Icanbewell web application would require frequent testing to ensure that the health information presented by the AI is sourced from credible, evidence-based resources and is free of misinformation or hallucinations [26].

Within the digital health literature, there are few studies evaluating general prevention apps such as Icanbewell. There is some research, however, focusing on evaluations of apps targeting the prevention of 1 specific condition. A Dutch qualitative study used focus groups to explore participants’ perceived barriers and facilitators to using a mobile health app on skin cancer screening. Interestingly, one of the main factors facilitating the use of mobile health among their participants was endorsement by health care providers and government regulatory bodies [27]. Similarly, a 2022 analysis of 267 COVID-19 apps found that government-developed apps were far more likely to be installed (incidence rate ratio [IRR] 8.16), rated, and commented on, although nongovernment apps received higher rating scores [28]. Implications of our work therefore include considering the integration of professional- and/or government-endorsed prevention-focused mobile health apps for the general Canadian population. Efforts to both create locally relevant, trustworthy, and accessible prevention information for the public and to support health care professionals in disseminating this evidence-based information are needed [29].

### Limitations

We acknowledge that our study had several limitations. Given the small convenience sample in our study, the results remain preliminary and exploratory. Our study’s self-selected sample was mostly made up of individuals who had a primary care provider and who were aged >50 years, which may not be representative of the general population that could benefit from the web application. Moreover, the Icanbewell web application may be more appealing to people who are already interested in health prevention, and therefore, those who responded to the survey may be people who are naturally more inclined to adopt healthy behaviors. Other studies of mobile health app users confirm this pattern in user characteristics, showing that most active users tend to: (1) come from high-income backgrounds with more years of education, (2) already rate their health highly, and (3) report healthy lifestyle behaviors at baseline [12,30,31]. We also recognize the underrepresentation of users who are unattached to health care providers, are younger, and are health care providers in our sample.

We also acknowledge that our choice of open-text questions to learn from users places some level of constraint on their ability to share their experiences. Specifically, this format did not allow follow-up probing and may have resulted in underspecified responses, potentially limiting the depth of themes identified in our inductive content analysis. Subsequent web application evaluations should prioritize obtaining richer knowledge from participants through semistructured interviews or focus groups on web application user experience, in which dialogue can be held.

Geographically, our study was led by a team based in Ottawa and disseminated primarily through Ottawa area clinical networks. Although we made efforts to extend recruitment to colleagues in other provinces and territories, a substantial proportion of respondents were from Ontario. Confirmation of results in a larger, more geographically diverse sample represents a priority for future work. Finally, due to an unforeseen technical error, the survey platform failed to track both the total number of users visiting the application web page and the number of incomplete, unsubmitted survey attempts. It was therefore impossible to calculate a standard response rate or a survey completion rate, which introduces an unknown risk of nonresponse and attrition bias.

Despite these limitations, several strategies were used to support the trustworthiness of our results in line with the criteria by Lincoln and Guba [24]. To support credibility and confirmability, 2 team members independently reviewed and discussed the survey data during analysis, allowing them to build consensus around emerging patterns. A third senior member then reviewed the data. For transferability, demographic characteristics of participants are reported (Table 1). In keeping with the exploratory, small sample nature of this study, no claims of broad generalizability are made. Dependability was achieved by maintaining consistent procedures for data review, interpretation, and documentation throughout the analysis.

### Future Directions

Future avenues for this work should use representative sampling and focus on obtaining the perspectives of groups underrepresented in this study to allow a more comprehensive and generalizable analysis of the user experience. It would be especially important to study the experience of individuals who are unattached to a health care provider to determine if such a web application may fill gaps in their prevention information needs [32].

Beyond this particular study, further research is needed to address the challenges associated with the sustainability of DHIs. This includes studying the effectiveness of DHIs based on actual health outcomes, encouraging further patient and clinician participation in DHI design and use, and integrating and regulating these DHIs within the health care system [6,33]^.^ The present study serves as a reminder that creating an all-encompassing web application dedicated to prevention that can keep up with Canada’s multijurisdictional, complex, and rapidly changing health care landscape is challenging. Likewise, securing funding to continuously update the content of such a web application is also challenging. Most importantly, planning for end-user engagement throughout the application development and implementation processes should be prioritized to maximize the benefit of the web application for users and ensure its usefulness and sustainability [33].

### Conclusions

This study evaluated the user experience of Icanbewell, a free, bilingual, pan-Canadian, evidence-based digital tool for prevention of disease. Our findings contribute to the growing body of evidence on the use of DHIs. User suggestions for improvement of this low-cost digital tool highlight several areas for priority in the development of an online Canadian health tool. Creating user-friendly navigation to enable individuals to access relevant information amid the overwhelming amount of Canadian health resources is paramount. Subsequent work should prioritize representative sampling to gather feedback from unattached individuals, younger populations, health care providers, and geographically diverse locations. Future involvement of patient partners in the design and evaluation of the web application is essential to ensure its inclusivity and relevance. Research on the effective delivery of free and reliable preventive health information to Canadians remains critical to combat misinformation and support the public through the ongoing primary care access crisis.

## Appendix

Multimedia Appendix 1 CHERRIES checklist.

Multimedia Appendix 2 Study pop-up window in English and French.

Multimedia Appendix 3 Survey questions in English and French.

Multimedia Appendix 4 Icanbewell web application analytics tracking gaps.

## Abbreviations

CHERRIES: Checklist for Reporting Results of Internet E-Surveys
DHI: digital health intervention
IRR: incidence rate ratio
