# Supplementary material for: User Experience of a Canadian Web Application for On-Demand Preventive Health Care: Cross-Sectional Online Survey Study
Source: JMIR Form Res. 2026 Oct 27;10:e90726. doi: 10.2196/90726 (PMC13752596; doi:10.2196/90726)
Supplement: Supplementary file 2 [file formative_v10i1e90726_app2.docx]

**Appendix 2 - Study Pop-up Window in English and French**

Figure S1a) English Pop-Up Window Announcement

Figure S1b) French Pop-Up Window Announcement
