# Supplementary material for: User Experience of a Canadian Web Application for On-Demand Preventive Health Care: Cross-Sectional Online Survey Study
Source: JMIR Form Res. 2026 Oct 27;10:e90726. doi: 10.2196/90726 (PMC13752596; doi:10.2196/90726)
Supplement: Supplementary file 3 [file formative_v10i1e90726_app3.docx]

**Appendix 3 - Survey Questions**

**English**

**Français**
