# Supplementary material for: User Experience of a Canadian Web Application for On-Demand Preventive Health Care: Cross-Sectional Online Survey Study
Source: JMIR Form Res. 2026 Oct 27;10:e90726. doi: 10.2196/90726 (PMC13752596; doi:10.2196/90726)
Supplement: Supplementary file 4 [file formative_v10i1e90726_app4.docx]

**Appendix 4 – Icanbewell app analytics tracking gaps**

Numbers of distinct users accessing the app during various time periods that we were able to document; missing data are due to unexpected data recording outages:

- July 3-9: 76 distinct users
- Aug 3-9: 42 distinct users
- Sept 2024: 79 distinct users
- Oct 2024: 111 distinct users
- Nov 2024: 63 distinct users
- Dec 2024: 41 distinct users
- Jan 2025: 40 distinct users
- Feb 2025: 26 distinct users
- March 2025: 48 distinct users
- April 2025: 33 distinct users
